# Comprehensive analysis of lncRNA expression profiles reveals a novel lncRNA signature to discriminate nonequivalent outcomes in patients with ovarian cancer

**DOI:** 10.18632/oncotarget.8653

**Published:** 2016-04-18

**Authors:** Meng Zhou, Yanying Sun, Yifan Sun, Wanying Xu, Zhaoyue Zhang, Hengqiang Zhao, Zhaohua Zhong, Jie Sun

**Affiliations:** ^1^ College of Bioinformatics Science and Technology, Harbin Medical University, Harbin, 150081, PR China; ^2^ Department of Microbiology, Harbin Medical University, Harbin, 150081, PR China

**Keywords:** long non-coding RNAs, ovarian cancer, outcome, BRCA1/2

## Abstract

There is growing evidence of dysregulated long non-coding RNAs (lncRNAs) serving as potential biomarkers for cancer prognosis. However, systematic efforts of searching for an expression-based lncRNA signature for prognosis prediction in ovarian cancer (OvCa) have not been made yet. Here, we performed comprehensive analysis for lncRNA expression profiles and clinical data of 544 OvCa patients from The Cancer Genome Atlas (TCGA), and identified an eight-lncRNA signature with ability to classify patients of the training cohort into high-risk group showing poor outcome and low-risk group showing significantly improved outcome, which was further validated in the validation cohort and entire TCGA cohort. Multivariate Cox regression analysis and stratified analysis demonstrated that the prognostic value of this signature was independent of other clinicopathological factors. Associating the outcome prediction with *BRCA1* and/or *BRCA2* mutation revealed a superior prognosis performance both in *BRCA1/2*-mutated and *BRCA1/2* wild-type tumors. Finally, a significantly correlation was found between the lncRNA signature and the complete response rate of chemotherapy, suggesting that this eight-lncRNA signature may be a measure to predict chemotherapy response and identify platinum-resistant patients who might benefit from other more efficacious therapies. With further prospective validation, this eight-lncRNA signature may have important implications for outcome prediction and therapy decisions.

## INTRODUCTION

Ovarian cancer (OvCa) is the most common malignant gynecologic cancer and remains the leading cause of cancer-related death in the western world [1]. In China, there is an increasing trend in the incidence of OvCa [2]. The aggressive surgical debulking followed by platinum-based chemotherapy is known as a standard treatment protocol for OvCa patients [3]. After first line treatment, despite response rates and complete responses were respectively > 80% and 40-60%, most patients eventually face relapse and the majority of them ultimately die of their recurrent disease resulting in an overall five-year survival probability less than 40% for patients with advanced OvCa [4, 5]. Therefore, there is an urgent need for prognostic markers that would be able to predict patients' poor outcome or chemotherapy resistance, and whether they will benefit from tailored treatment strategies.

Long non-coding RNAs (lncRNAs), are the largest class of non-coding RNAs (ncRNAs) with a length of more than 200 base-pairs [6]. Although most of lncRNAs have not been functionally characterized, there is increasing evidence suggesting that lncRNAs may contribute to a significant layer of genome regulatory information by negatively or positively regulating gene expression at the transcriptional, post-transcriptional and epigenetic levels [7–9]. More and more studies have demonstrated that lncRNAs play important roles in a variety of biological processes [9–11], and their dysregulation and mutation have been implicated in many complex diseases, including cancers [12–18]. Large-scale transcriptional profile analyses have reported the highly aberrant lncRNA expression across multiple human cancer types in the tissue- and cancer type-specific manners [19–22]. Some well-known cancer-associated lncRNAs, such as *H19* [23], *XIST* [24], *HOTAIR*[25], *MALAT1*, *MEG3* [26], *HNF1A-AS1* [27] and *PVT1* [28], have indicated oncogenic and/or tumor suppressive roles like protein-coding genes in various cancers. As lncRNAs do not code for proteins, lncRNA expression may be a better indicator of the tumor status, implying the potential and superiority as independent biomarkers for early diagnosis and prognosis prediction in cancers [29]. Currently, several expression-based lncRNA signature have been identified to predict patients' survival in some cancers, including glioblastoma multiforme [30], oesophageal squamous cell carcinoma [31], colorectal cancer [32], breast cancer [33–35], lung cancer [36] and multiple myeloma [37]. Recent studies have revealed that changes in lncRNA expression were associated with OvCa tumorigenesis and metastasis. For example, lncRNAs *UCA1* and *HOTAIR* are overexpressed in epithelial ovarian cancer (EOC) associated with a worse prognosis [38, 39]. Three lncRNAs (*TC0100223*, *TC0101686* and *TC0101441*), which showed differential expression between ERα-positive and ERα-negative EOC tissues, were associated with poor prognosis of ERα-positive EOC patients [40]. Some differentially expressed lncRNAs were identified in paired high and low metastatic OvCa cells [41]. A recent study of lncRNA *AB073614* showed that patients with higher *AB073614* expression had poor overall survival in OvCa [42].

In this study, we performed a comprehensive analysis for lncRNA expression profiles and clinical outcome of a large number of OvCa patients from The Cancer Genome Atlas (TCGA) Research Network to investigate whether lncRNA expression profiling could be used as a prognostic signature for accurately prognosticating clinical outcome and chemotherapy response in patients with OvCa.

## RESULTS

### Identification of prognostic lncRNAs associated with outcome in patients with OvCa

To detect potential prognostic lncRNAs, we subjected the expression data of each lncRNA in the training cohort to univariate Cox proportional hazards regression analysis with overall survival as the dependent variable. A total of eight lncRNAs were identified as potential prognostic lncRNAs that were significantly correlated with overall survival (p<0.001) (Table 1). Among these prognostic lncRNAs, six lncRNAs having negative coefficients were shown to be protective lncRNAs whose high expression levels were associated with longer survival. The remaining two lncRNAs had positive coefficients and were risky lncRNAs whose high expression levels were associated with shorter survival.

**Table 1:** The detailed information of eight prognostic lncRNAs significantly associated with overall survival in patients with OvCa

Ensemble ID	Gene name	Chromosome (GRCh38)	P-value^a^	Hazard ratio^a^	Coefficient^a^
ENSG00000236244	*RP4-799P18.3*	Chr 1: 234,268,583-234,272,500 (−)	4.0e-05	0.73	−0.28
ENSG00000225706	*PTPRD-AS1*	Chr 9: 8,858,130-8,862,255 (+)	1.23e-04	1.35	0.27
ENSG00000259331	*RP11-57P19.1*	Chr 15: 94,600,014-94,600,821(+)	4.7e-04	0.71	−0.21
ENSG00000232093	*RP11-307C12.11*	Chr 1: 155,045,191-155,046,118(−)	5.56e-04	0.65	−0.24
ENSG00000250551	*RP11-254I22.1*	Chr 5: 96,050,115-96,215,519(+)	5.81e-04	0.61	−0.45
ENSG00000240996	*RP11-80H5.7*	Chr 10: 89,694,295-89,697,928 (−)	6.15e-04	1.32	0.25
ENSG00000261071	*RP1-223E5.4*	Chr 6: 13,614,111-13,615,155 (−)	7.45e-04	0.64	−0.30
ENSG00000236289	*GACAT3*	Chr 2: 16,050,427-16,085,801(+)	9.32e-04	0.48	−0.11

a Derived from the univariable Cox proportional hazards regression analysis in 263 patients of training cohort.

### Acquisition of an eight-lncRNA prognostic signature from the training cohort

**Figure 1:**
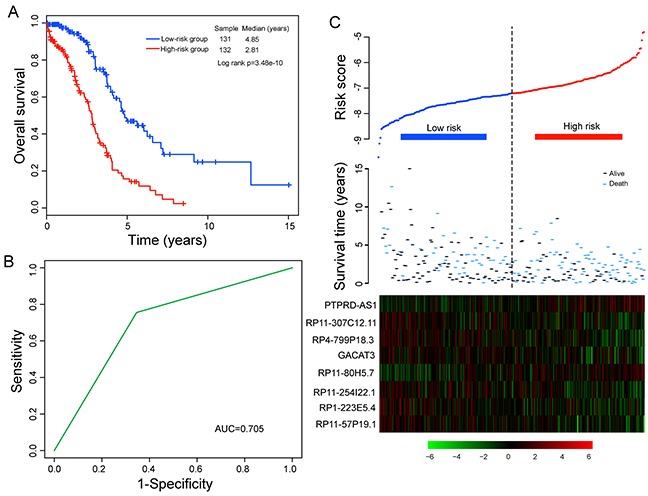
Association between the eight-lncRNA expression signature and overall survival of patients in the training cohort **A.** Kaplan-Meier survival curves of overall survival between high-risk and low-risk patients. **B.** Time-dependent ROC curves analysis for survival prediction by the eight-lncRNA signature within 5 years as the defining point. **C.** LncRNA risk score analysis of patients in the training cohort.

To evaluate the relative contribution of these eight prognostic lncRNAs for survival prediction when considering interrelationship among them, we performed a multivariate Cox regression analysis for these eight prognostic lncRNAs with overall survival as a dependent variable. Then, we developed a prognostic model by the risk scoring method as previously described [43, 44] for survival prediction based on the expression levels of these eight lncRNAs and their relative contributions derived from above multivariate analysis as follows: Risk score= (−0.28051 × expression value of *RP4-799P18.3*) + (0.270976 × expression value of *PTPRD-AS1*) + (−0.211224× expression value of *RP11-57P19.1*) + (−0.239482 × expression value of *RP11-307C12.11*) + (−0.445144 × expression value of *RP11-254I22.1*) + (0.245869 × expression value of *RP11-80H5.7*) + (−0.295698 × expression value of *RP1-223E5.4*) + (−0.114835× expression value of *GACAT3*). This eight-lncRNA signature-based prognostic model assigned a risk score for each patient. Using the median risk score as the cutoff (−7.203), patients of the training cohort were divided into high-risk group (n=132) and low-risk group (n=131). The patients in the low-risk group were expected to have better survival outcomes. As a result, patients in the low-risk group had significantly longer median overall survival than those in the high-risk group (median 4.85 years versus 2.81 years; p=3.48e-10, log-rank test) (Figure 1A). The overall survival rate of patients at five years in the low-risk group was 46.9%, whereas the corresponding rate in the high-risk group was 15.8%. The univariate analysis revealed a significant association between the risk score and overall survival, in which the hazard ratio (HR) of high-risk group versus low-risk group for overall survival was 3.12 (95% confidence interval (CI) =2.15-4.53; p=2.22e-09) (Table 2). The time-dependent ROC curves analysis for eight-lncRNAs signature-based prognostic model achieved an area under the curve (AUC) of 0.705 at five years (Figure 1B). These results demonstrated better performance of this eight-lncRNA signature in prognosis prediction of patients with OvCa. Figure 1C showed the risk score distribution, survival status and lncRNA expression of 263 patients in the training cohort, ranked according to the risk scores of the eight-lncRNA signature. We found that patients with high-risk scores tended to express two risky lncRNAs, and patients with low-risk scores tended to express six protective lncRNAs (Figure 1C).

**Table 2:** Univariate and multivariate Cox regression analysis of overall survival in each cohort

Variables	Univariate analysis			Multivariate analysis		
	HR	95% CI of HR	P-value	HR	95% CI of HR	P-value
Training cohort (n=263)						
Eight-lncRNA signature risk score						
Low	1 (reference)			1 (reference)		
High	3.12	2.15-4.53	2.22e-09	2.37	1.53-3.67	1.07e-04
Age	1.03	1.01-1.04	1.73e-03	1.02	1.00-1.04	4.21e-02
Stage						
II	1 (reference)			1 (reference)		
III	4.33	1.37-13.66	1.25-e02	23481381	0.00-Inf	0.99
IV	5.89	1.75-19.82	4.18e-03	32442060	0.00-Inf	0.99
Grade						
G1/G2	1 (reference)			1 (reference)		
G3/G4	1.17	0.78-1.77	0.45	1.33	0.83-2.14	0.24
Residual						
0-10 mm	1 (reference)			1 (reference)		
>10 mm	0.97	0.65-1.45	0.88	0.76	0.49-1.16	0.20
Validation cohort (n=281)						
Eight-lncRNA signature risk score						
Low	1 (reference)			1 (reference)		
High	1.40	1.01-1.92	4.02e-02	1.50	1.05-2.15	2.71e-02
Age	1.02	1.00-1.03	2.32e-02	1.03	1.01-1.04	2.98e-03
Stage						
II	1 (reference)			1 (reference)		
III	1.34	0.49-3.63	0.57	1.26	0.39-4.02	0.70
IV	1.84	0.65-5.24	0.25	1.95	0.58-6.56	0.28
Grade						
G1/G2	1 (reference)			1 (reference)		
G3/G4	1.92	1.01-3.67	4.69e-02	2.66	1.21-5.85	1.47e-02
Residual						
0-10 mm	1 (reference)			1 (reference)		
>10 mm	1.28	0.88-1.86	0.21	1.10	0.74-1.62	0.65
TCGA cohort (n=544)						
Eight-lncRNA signature risk score						
Low	1 (reference)			1 (reference)		
High	2.00	1.57-2.55	1.71e-08	1.81	1.38-2.38	2.11e-05
Age	1.02	1.01-1.03	1.17e-04	1.02	1.01-1.04	4.28e-04
Stage						
II	1 (reference)			1 (reference)		
III	2.65	1.25-5.62	1.13e-02	2.27	0.72-7.14	0.16
IV	3.55	1.61-7.83	1.70e-03	3.35	1.03-10.90	4.45e-02
Grade						
G1/G2	1 (reference)			1 (reference)		
G3/G4	1.39	0.998-1.94	5.12e-02	1.72	1.17-2.53	6.25e-03
Residual						
0-10 mm	1 (reference)			1 (reference)		
>10 mm	1.07	0.82-1.40	0.62	0.97	0.73-1.28	0.82

Abbreviations: HR, hazard ratio; CI, confidence interval.

In both univariate and multivariate Cox regression analysis, age was evaluated as continuous variable.

### Confirmation of the eight-lncRNA signature for survival prediction in the validation cohort and entire TCGA cohort

**Figure 2:**
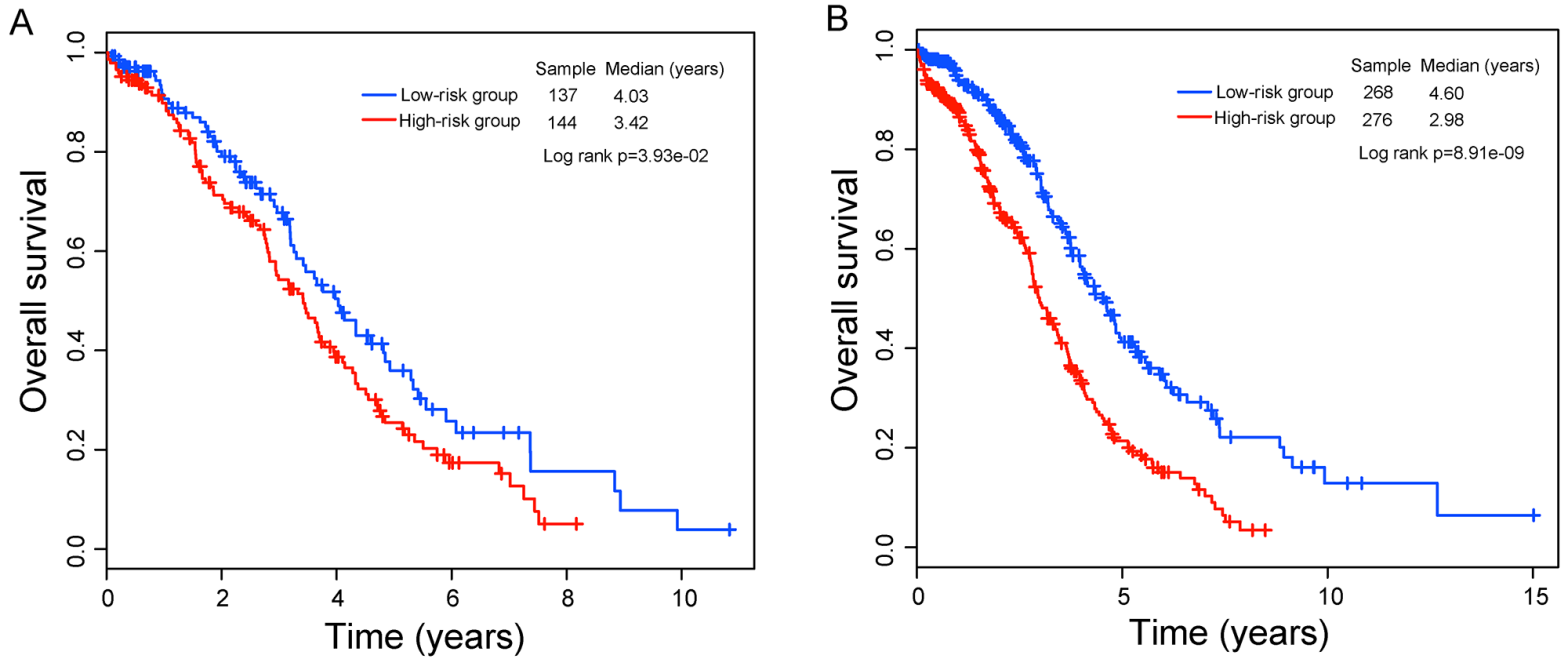
Kaplan–Meier curves analysis of overall survival between high-risk and low-risk patients **A.** The validation cohort. **B.** The entire TCGA cohort.

The prognostic power of the eight-lncRNA signature in survival prediction was further tested in the validation cohort. By using the same risk score model and cutoff value deriving from the training cohort, 281 patients of the validation cohort were classified into either high-risk group (n=144) or low-risk group (n=137). In consistence with our findings in the training cohort, Kaplan-Meier analysis using this eight-lncRNA signature showed significant difference in their overall survival between high-risk group and low-risk group (p=3.93e-02, log-rank test) (Figure 2A). Patients in the low-risk group have a significantly longer median overall survival than those in the high-risk group (median 4.03 years versus 3.42 years). In univariate analysis, the HR of high risk scores versus low-risk scores for overall survival was 1.40 (95% CI=1.01-1.92; p=4.02e-02) (Table 2), demonstrating a significant association between risk scores and patients' overall survival. The five-year survival rate of the low-risk group was 36%, whereas the corresponding rate in the high-risk group was 25.5%. Validation of the eight-lncRNA signature in the validation cohort of 281 patients produced an ROC with an AUC of 0.555 at five years (see Supplementary Figure S1A online).

When this eight-lncRNA signature was further applied to the entire TCGA OvCa cohort (combining the training and validation cohorts), similar risk stratification results were observed. As in the training and validation cohorts, this eight-lncRNA signature was able to stratify 544 patients of the entire TCGA cohort into the high-risk group (n=276) and low-risk group (n=268) with significantly different survival (median 2.98 years versus 4.6 years; p=8.91e-09, log-rank test) (Figure 2B). At five years, the respective absolute difference in overall survival between the low-risk group and high-risk group was 19.9% (41.3% versus 21.4%). The AUC of time-dependent ROC curves for the eight-lncRNA signature in the entire TCGA cohort was 0.609 at five years (see Supplementary Figure S1B online).

The distribution of risk scores, survival status and expression levels of these eight lncRNAs in the testing and entire TCGA cohorts were shown in Supplementary Figure S2 online, which yielded similar results observed in the training cohort.

### Independence of prognostic value of the eight-lncRNA signature from other clinical variables

**Figure 3:**
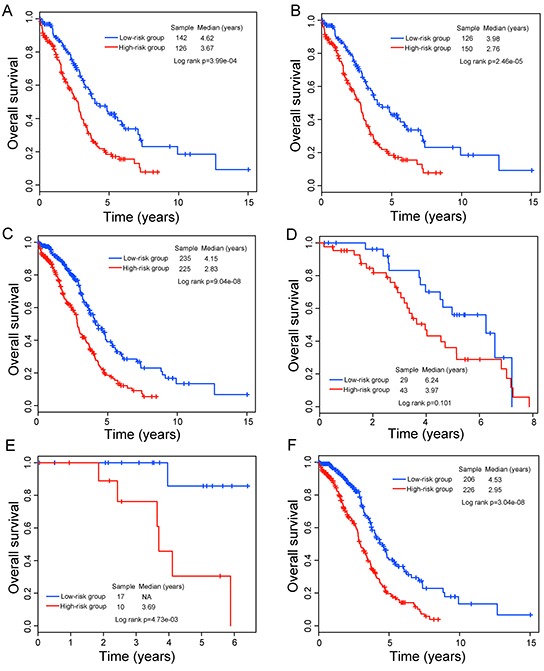
Survival prediction in TCGA patients stratified by age, grade and stage **A.** Kaplan-Meier curves for younger TCGA patients. **B.** Kaplan-Meier curves for elder TCGA patients. **C.** Kaplan–Meier curves for high-grade TCGA patients. **D.** Kaplan–Meier curves for low-grade TCGA patients. **E.** Kaplan-Meier curves for stage II TCGA patients. **F.** Kaplan–Meier curves for stage III TCGA patients.

To determine whether the prognostic value of the eight-lncRNA signature is independent of other clinical variables, we performed multivariate Cox regression analysis in each patient cohort including risk scores, age, tumor stage, tumor grade and surgical debulking status as covariables. The results from the training cohort showed that lncRNA signature-based risk scores (HR=2.37, 95% CI=1.53-3.67; p=1.07e-04) and age (HR=1.02, 95% CI=1.0-1.04; p=4.21e-02) were significantly correlated with survival of patients with OvCa. Specifically, the eight-lncRNA signature still maintained a significant correlation with survival when adjusting for age, tumor stage, tumor grade and surgical debulking status in the validation cohort and entire TCGA cohort. The HR of high-risk group versus low-risk group for overall survival was 1.5 in the validation cohort (95% CI=1.05-2.15; p=2.71e-02), as well as in the entire TCGA cohort (HR=1.81, 95% CI=1.38-2.38; p=2.11e-05) when controlling for other clinical variables (Table 2). However, we found that two clinical variables, age and tumor grade, were significantly associated with overall survival in at least two of three patient cohorts. So we further performed data stratification analysis according to age and tumor grade. First, all patients were stratified into a younger stratum (n=268) and an elder stratum (n=276) according to the median age (59 years old). The eight-lncRNA signature could subdivide younger patients into high-risk group (n=126) and low-risk group (n=142). The median overall survival time of patients in the low-risk group was significantly longer than that of patients in the high-risk group (median 4.62 years versus 3.67 years; p=3.99e-04, log-rank test) (Figure 3A). Similarly, among patients within the elder stratum, the same prognostic model was able to separate patients into two risk subgroups with significantly different survival (median 2.76 years versus 3.98 years; p=2.46e-05, log-rank test) (Figure 3B). Then all patients were further stratified into low-grade (G1/G2) and high-grade subgroups (G3/G4) according to tumor grade. The results of stratified analysis showed effective prognostic power both in the low-grade and in the high-grade patient subgroups. The patients with high-grade were divided into either a high-risk group (n=225) with shorter survival or a low-risk group (n=235) with longer survival (median 2.83 years versus 4.15 years; p=9.04e-08) (Figure 3C). Similar results were observed for low-grade patient subgroup, in which patients were classified into two risk subgroups with marginally significantly different survival time (median 3.97 years versus 6.24 years; p=0.101, log-rank test) (Figure 3D).

Risk evaluation for newly diagnosed patients at the early tumor stage will improve adjuvant treatment decisions making it possible to identify high-risk patients who might benefit from adjuvant therapy. Therefore, separate validation of this eight-lncRNA signature in survival prediction was performed for stage II and III patients. Within each stage stratum, patients were classified as high-risk and low risk according to the same prognostic model and risk score cutoff. The Kaplan-Meier analysis showed that patients with high-risk scores tended to have shorter survival than those with low-risk scores (median 3.69 years versus >5 years; p=4.73e-03, log-rank test for stage II patients and median 2.95 years versus 4.53 years; p=3.04e-08, log-rank test for stage III patients) (Figure 3E and 3F). Taken together, the results of multivariate analysis and stratification analysis demonstrated that the prognostic value of the eight-lncRNA signature is independent of other clinical variables for survival prediction of patients with OvCa.

### Ability of the eight-lncRNA signature to discriminate *BRCA1/2*-mutated or *BRCA 1/2* wild-type tumors

**Figure 4:**
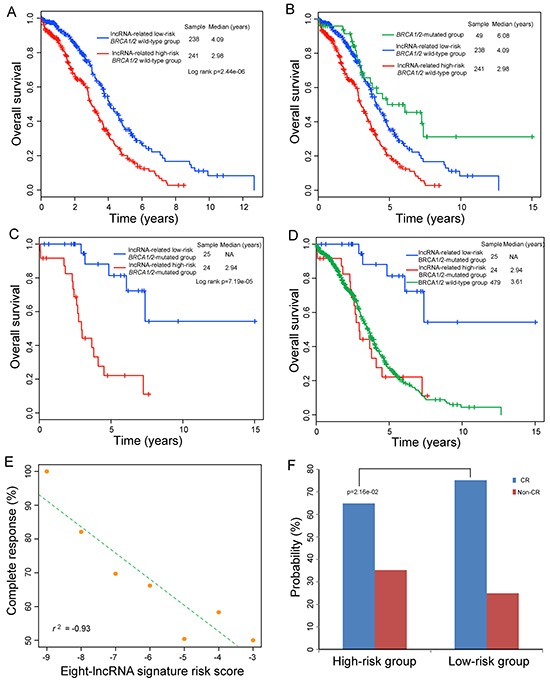
Relationship between the eight-lncRNA expression signature and clinical outcome in *BRCA1/2*-mutated or *BRCA 1/2* wild-type OvCa tumors **A.** Kaplan-Meier curves analysis of overall survival between lncRNA-related high-risk and low-risk *BRCA 1/2* wild-type patients. **B.** Differences in overall survival were assessed among the three groups. The log-rank p value of overall survival for the lncRNA-related high-risk *BRCA1/2* wild-type group versus *BRCA1/2*-mutated group is 1.19e-05 and the log-rank p value of overall survival for the lncRNA-related *BRCA1/2* wild-type low-risk group versus *BRCA1/2*-mutated group is 4.79e-02. **C.** Kaplan-Meier curves analysis of overall survival between lncRNA-related high-risk and low-risk *BRCA1/2*-mutated patients. **D.** Differences in overall survival were assessed among the three groups. The log-rank p value of overall survival for the lncRNA-related low-risk *BRCA1/2*-mutated group versus *BRCA 1/2* wild-type group is 3.28e-06 and the log-rank p value of overall survival for the lncRNA-related high-risk *BRCA1/2*-mutated group versus *BRCA 1/2* wild-type group is 0.749. **E.** Correlation of the eight-lncRNA signature with complete response. The Pearson correlation coefficient was calculated to assess the relationship between the eight-lncRNA signature and the likelihood of complete response. The straight line depicts the least squares linear regression line through the data points. **F.** Differences in complete response ratios between high-risk group and low-risk group.

Previous studies have suggested that *BRCA1* and *BRCA2* mutations are associated with clinical outcome of OvCa patients, and patients harboring *BRCA1* and/or *BRCA2* mutation (hereafter inferred as *BRCA1/2* mutation) subjected to platinum-based treatment have an improved clinical outcome compared with *BRCA 1/2* wild-type patients. Therefore, we further assessed the prognostic value of the eight-lncRNA signature for the patients with or without *BRCA1/2* mutation by stratification analysis, which stratified patients into *BRCA1/2*-mutated group and *BRCA 1/2* wild-type group. Using the same score formula, we classified patients in *BRCA 1/2* wild-type group into a high-risk group (n=241) and a low-risk group (n=238) using the same cutoff as in the training set. Patients in the low-risk group had significantly longer survival time than those in the high-risk group (median 4.09 years versus 2.98 years; p=2.44e-06; log-rank test) (Figure 4A). Then we performed pairwise comparisons of overall survival between lncRNA-related high-risk *BRCA 1/2* wild-type group, lncRNA-related low-risk *BRCA 1/2* wild-type group and *BRCA1/2*-mutated group. This analysis showed that patients in the lncRNA-related high-risk *BRCA 1/2* wild-type group had significantly shorter survival time than did patients in the *BRCA1/2*-mutated group (median 2.98 years versus 6.08 years; p=1.19e-05) and lncRNA-related low-risk *BRCA 1/2* wild-type group (median 2.98 years versus 4.09 years; p=2.44e-06, log-rank test) (Figure 4B). However, *BRCA1/2*-mutated patients only showed marginally significant difference in clinical outcome from patients in the lncRNA-related low-risk *BRCA 1/2* wild-type group (median 6.08 versus 4.09 years; p=4.79e-02). These results suggested that although *BRCA 1/2* wild-type patients did not harbor *BRCA1/2* mutation, a substantial subset of *BRCA 1/2* wild-type patients with lncRNA-related low-risk scores still had a good prognosis and could benefit platinum-based chemotherapy.

Then forty-nine OvCa patients with *BRCA1* mutation and/or with *BRCA2* mutation were stratified into the *BRCA1/2*-mutated group. We found that patients of *BRCA1/2*-mutated group also could be separated into those likely to have good outcome and those likely to have poor outcome according to the risk scores of the eight-lncRNA signature. The survival time of *BRCA1/2*-mutated patients in the high-risk group (n=24) was significantly lower than that of *BRCA1/2*-mutated patients in the low-risk group (n=25) (median 2.94 years >7.38 years; p=7.19e-05, log-rank test) (Figure 4C). Furthermore, *BRCA1/2*-mutated patients in the low-risk group had significantly longer survival time than the wild-type patients (median >7.38 years versus 3.61 years; p=3.28e-06, log-rank test), whereas those in the high-risk group showed no significant difference in clinical outcome from the wild-type patients (median 2.94 years versus 3.61 years; p=0.749, log-rank test) (Figure 4D), which supported previous finding that not all *BRCA*-mutated patients exhibit favorable clinical outcome [45].

Based on above observations, we further explored whether there was an association between risk score of the eight-lncRNA signature and the likelihood of complete response (CR). 437 OvCa patients with CR or Non-CR information were analyzed. We first plotted the percentage of OvCa patients achieving CR as a function of the risk score, and observed that the probability of OvCa patients achieving CR was significantly correlated with risk score of this eight-lncRNA signature (Pearson correlation coefficient *r*^2^=−0.93, p=2.69e-03) (Figure 4E). Patients with low-risk scores tended to have high likelihood of CR and those with high-risk scores had low likelihood of CR. In detail, 75.11% of patients in the low-risk group achieved CR, whereas 64.81% of patients in the high-risk group achieved CR (p=2.16e-02, Fisher exact test) (Figure 4F), implying that the risk score of the eight-lncRNA signature had the potential to reflect the sensitivity to platinum therapy. Therefore, these results suggested that *BRCA*-mutated patients with lncRNA-related high-risk scores may be platinum resistant and need individual appropriate treatment strategies except for platinum-based chemotherapy. This is consistent with previous studies showing nearly 40% of *BRCA*-mutated OvCa tumors exhibited platinum resistance and experienced multiple cycles of non-beneficial toxic chemotherapy leading to a poor clinical outcome [46, 47].

### Functional characteristics of prognostic lncRNAs

**Figure 5:**
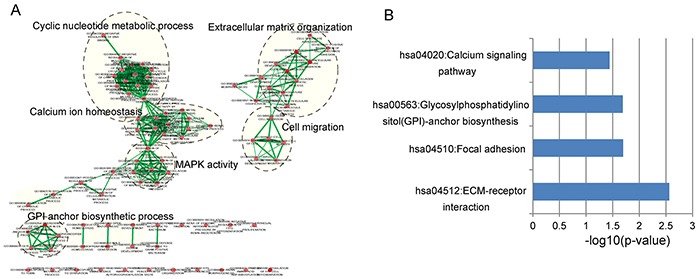
Functional enrichment analysis of protein-coding gene co-expressed with eight prognostic lncRNAs **A.** The functional map of enriched GO terms with each node indicates an enriched GO term and each edge represents the common genes shared between connecting enriched GO terms. **B.** Significantly enriched KEGG pathways.

We first obtained genome-wide protein-coding gene (PCG) expression profiles in 544 OvCa patients from Du's study [48], including 18292 PCGs. lncRNAs do not encode proteins and their functions are known to be associated with co-expressed PCGs [49, 50]. Therefore, we examined the expression correlation between lncRNAs and PCGs using paired lncRNA and PCG expression profiles in 544 OvCa patients. Only those PCGs with a Pearson correlation coefficients ranked in the top 1% for each prognostic lncRNA were considered as lncRNA-correlated PCGs. To explore potential functional roles of these eight prognostic lncRNAs in OvCa development and progression, we performed functional enrichment analysis of lncRNA-correlated PCGs. The results from GO enrichment analysis revealed that lncRNA-correlated PCGs were significantly enriched in 87 GO terms, in which functionally related GO terms could be organized into six functional clusters including cyclic nucleotide metabolic process, extracellular matrix (ECM) organization, calcium ion homeostasis, cell migration, MAPK activity and GPI anchor biosynthetic process (Figure 5A). KEGG pathway enrichment analysis of lncRNA-correlated PCGs showed that four KEGG pathways, including ECM-receptor interaction, Focal adhesion, GPI-anchor biosynthesis and Calcium signaling pathway were significantly enriched (Figure 5B). These enriched GO function and KEGG pathway of lncRNA-correlated PCGs have been reported to be involved with OvCa through our literature review. The functional analysis of lncRNAs suggested that these prognostic lncRNAs might participate in OvCa tumorigenesis through positively regulating lncRNA-related PCGs to affect known OvCa-related biological pathways.

## DISCUSSION

In terms of the heterogeneity of clinical outcomes in OvCa patients, there is a critical need for reliable prognostic factors pinpointing a subset of patients with poor prognosis who would therefore benefit from additional treatment options. However, traditional clinicopathological prognostic markers, such as age, stage, debulking status after primary surgery and response to chemotherapy are not satisfactory for prognosis prediction and treatment decisions of individual patient. Recent large-scale genomic analyses have revealed a catalogue of molecular characteristics associated with OvCa outcome and therapeutic treatment [51]. Molecular expression profiles have been used to identify outcome-related molecular signatures which provided important implication for prognosis prediction and molecular mechanism of OvCa [52–58]. However, most existing studies have focused on mRNA and microRNA expression. Knowledge is now rapidly emerging on the functional roles of lncRNAs in cancer initiation and progression, representing a significant untapped molecular resource for cancer prognosis and treatment.

In this study, we conducted a comprehensive analysis of lncRNA expression profiles in a large number of OvCa patients from TCGA, and identified an eight-lncRNA signature predictive of overall survival using the sample-splitting method and Cox regression analysis. We found that six lncRNAs were correlated with good survival and two lncRNAs were correlated with poor survival. As previously described [59, 60], three methods (independent dataset test, subsampling test and jackknife test) were widely used to examine the accuracy of predictive model. Therefore, we validated the predictive power of this eight-lncRNA signature on an independent and non-overlapping set of 281 TCGA patients as well as on the entire TCGA cohort. Furthermore, the eight-lncRNA signature is independent of other clinicopathological factors, including age, stage, grade and surgical debulking status. It is well known that younger women with OvCa show better survival rates than older women. Statistics showed that younger women with OvCa were treated more aggressively than older women, and most of younger patients received chemotherapy [61]. Although treatment combination of surgery plus chemotherapy increases the survival rates for younger patients, chemotherapy is accompanied by cumulative side effects that affect patients' life quality. Therefore, further stratification of younger women with OvCa who might or might not benefit from adjuvant chemotherapy is urgently needed. In the stratified analysis, the eight-lncRNA signature showed prognostic value both in younger and older patients. The eight-lncRNA signature could subdivide patients in the same age stratum into high-risk and low-risk groups with significantly different clinical outcome.

Previous studies have suggested that homologous recombination (HR) deficiency can bring about error-prone repair of DNA double-strand breaks (DSBs) and are used to identify platinum-sensitive tumors [47, 62]. However, defining HR deficiency is still a challenge. It is suggested that *BRCA1* and *BRCA2* mutations are associated with HR deficiency in OvCa, therefore OvCa patients harboring *BRCA1/2* mutations subjected to platinum-based treatment have favorable outcomes compared with *BRCA 1/2* wild-type patients. Recent studies found that some of *BRCA 1/2* wild-type patients in OvCa may also harbor HR deficiency and have significantly improved survival when subjected to platinum-based treatment. Therefore, we applied this signature to test whether this eight-lncRNA signature was able to identify those *BRCA1/2* wild-type patients who will benefit from platinum-based chemotherapy. Survival analysis of 479 *BRCA1/2* wild-type patients demonstrated that this eight-lncRNA was able to identify a subgroup of patients with wild-type *BRCA1/2* with a remarkably good clinical outcome. Further analysis found that even for *BRCA*-mutated patients, this eight-lncRNA signature also revealed a prognostic value which stratified *BRCA*-mutated patients into cases of significantly improved outcome and cases of poor outcome. This is consistent with recently published results showing a fraction of *BRCA*-deficient tumors were resistant to chemotherapy [63, 64]. With further validation, a closely association between the risk score of eight-lncRNA signature and CR was observed, implying that this eight-lncRNA signature may be a measure to predict chemotherapy response and be used to identify platinum-resistant patients who might benefit from other more efficacious therapies.

Until now, more than tens of thousands of lncRNAs have been discovered and recorded in several publicly biological databases, such as GENCODE [65], NONCODE [66] and LNCipedia [67]. However, only a very few lncRNAs were well functionally characterized. By reviewing literatures, only one of eight prognostic lncRNAs, *GACAT3*, has been reported to play crucial roles in the gastric carcinoma [68]. Previous studies have suggested that lncRNAs participated in biological processes by interacting with PCGs involved in the same processes, making it possible to infer lncRNA function from their co-expressed PCGs [49, 69, 70]. Therefore, we performed functional enrichment analysis for co-expressed PCGs to uncover potential biological processes lncRNAs involved in. We found that these eight prognostic lncRNAs might participate in several biological processes, including ECM-receptor interaction, Focal adhesion, MAPK activity, GPI-anchor biosynthesis and Calcium signaling pathway. Proteomic analyses have demonstrated that aberrant expression of key members in ECM and Focal adhesion pathways are associated with invasive behavior by ovarian cancer cells [71]. MAPK pathways can control fundamental cellular processes by linking extracellular signals to the machinery, and distinct groups of MAPK pathways have been widely studied, revealing important roles of MAPK pathways in cancers, including OvCa [72, 73]. Glycosylphosphatidylinositol (GPI) anchor is an unique type of glycoconjugate, and abnormal expression levels of certain components in the GPI-anchor biosynthetic pathway have been reported to be associated with various cancers [74]. A recent experimental study provided evidence for the role of calcium-related genes in mediating cisplatin resistance in ovarian cancer cells [75]. Taken together, these analyses suggested that the eight prognostic lncRNAs might have important biologic relevance in regulating or interacting PCGs involved in OvCa, but further experimentally validation is required. As demonstrated in previous studies [59, 76, 77], we shall make efforts in our future work to provide a publicly accessible web-server for our predictive model.

In conclusion, we succeeded in identifying and validating an eight-lncRNA signature for prognosis prediction in OvCa patients by performing genome-wide analysis of lncRNA expression profiles in a large cohort of TCGA patients, which was able to classify patients into high-risk group showing poor outcome and low-risk group showing significantly improved outcome. The eight-lncRNA signature maintained independent prognostic value in multivariate and stratified analysis, controlling for other known prognostic factors such as age, stage, grade, debulking status and *BRCA1/2* mutation status. Moreover, the lncRNA signature was significantly correlated with the response to chemotherapy. To our knowledge, the lncRNA expression profiles and OvCa patients derived from TCGA are unprecedented in comprehensiveness and in size, and there is no other available independent datasets to validate our findings. When our study was in progress, two novel immune-associated lncRNAs (*RP11-284N8.3.1* and *AC104699.1.1*) were identified to predict survival of patients with different OvCa stages by using lncRNA-mRNA co-expression network methods [78]. Our study, taken together with Guo's study, highlighted important implications of lncRNAs as novel biomarkers for outcome prediction and therapy decisions.

## MATERIALS AND METHODS

### Patient dataset

544 patients with serous ovarian carcinoma (stages II, III and IV) and their related clinical information were obtained from TCGA data portal (https://tcga-data.nci.nih.gov/tcga/). The TCGA OvCa patient cohort was divided into a training cohort (batches 18-40) and a validation cohort (batches 9-17), which results in a 263-sample training cohort and a 281-sample validation cohort. Detailed clinical information of OvCa patients enrolled in this study, including age, tumor stage, tumor grade, response to chemotherapy, and surgical debulking, was listed in Table 3 and Supplementary Table S1. The somatic and germline mutation information of *BRCA1* and *BRCA2* genes from whole exome sequencing was downloaded from the cBioPortal Cancer Genomics (http://www.cbioportal.org/) [79].

**Table 3:** Tumor characteristics of ovarian cancer patients in this study

Characteristic	Training cohort (batches 18-40, n=263)	Validation cohort (batches 9-17, n=281)	TCGA cohort (n=544)
Age	59.02±11.77	60.11±11.29	59.58±11.53
Vital status			
Alive	136	120	256
Dead	127	161	288
Stage^a^			
II	19	8	27
III	210	222	432
IV	34	51	85
Grade^b^			
G1-G2	55	17	72
G3-G4	201	259	460
Response to therapy			
CR	138	168	306
Non-CR	63	68	131
Residual tumor size			
0-10 mm	99	147	246
>10 mm	79	60	139

Numbers do not sum due to the back of data of interest.

a Stage based on International Federation of Gynecology and Obstetrics (FIGO)

b Grade based on histological features.

c CR depicts Complete Response and Non-CR depicts non-complete response, including partial response, stable disease and progressive disease.

### Acquisition of lncRNA expression profiles of OvCa patients

Genome-wide lncRNA expression profiles of patients with OvCa were obtained from Du's study by repurposing the probes from Affymetrix Human Exon 1.0 ST microarray [48]. Briefly, the probe sets of Affymetrix Human Exon 1.0 ST microarray were re-mapped to human genome (hg 19), protein-coding transcripts, pseudogene transcripts and lncRNA sequences. Those probes that mapped to lncRNA sequences uniquely and perfectly were kept to represent lncRNAs. The lncRNA expression levels were obtained by calculating the background-corrected intensity of all probes mapped to this lncRNA. To account for the heterogeneity of different biological samples and different batches in systematic measurement, the expression value of lncRNA was standardized using quantile-normalized method and an empirical Bayes method [80]. Then the lncRNAs derived from microarray re-annotation and lncRNAs from GENCODE project (http://www.gencodegenes.org/, release 23) [65] were cross-reference by Ensembl id and gene name to reduce redundant and inaccurate annotations. Finally, we obtained expression profiles of 7952 lncRNAs in 544 OvCa patients.

### Construction of lncRNA-based prognostic signature

A univariate Cox regression analysis was performed to examine the association between expression levels of lncRNAs and patients' overall survival in the training cohort. Those lncRNAs with p-value <0.001 were selected as predictive lncRNAs whose expression levels were significantly associated with patients' overall survival. In order to evaluate relative contribution of predictive lncRNAs for survival prediction, they were fitted in a multivariate Cox regression analysis with overall survival as the dependent variable. A lncRNA expression-based prognostic risk score model was constructed by the linear combination of the expression levels of predictive lncRNAs with the multivariate Cox regression coefficient as the weight. This lncRNA prognostic model could calculate an lncRNA expression-based risk score for each patient and classify patients into high-risk group and low-risk group using the median risk score from the training cohort.

### Statistical analysis

Kaplan-Meier survival curves were used to estimate overall survival time for patients with predicted high- or low-risk scores, and the survival differences between high-risk group and low-risk group were assessed by a two-sided log-rank test using the R package “survival” [81]. Multivariate analyses were performed using Cox proportional hazards regression model to determine whether the lncRNA prognostic model was independent of other clinical variables, adjusting for age, tumor stage, grade, surgical debulking status, and risk scores. Hazard ratio (HR) and 95% confidence intervals (CI) were estimated by Cox proportional hazards regression model. The time-dependent receiver operating characteristic (ROC) curve analysis within 5 years as the defining point was preformed using the R package “survivalROC” [82], which has been widely used to assess the predictive accuracy of prognostic model or markers for time dependent disease outcomes [83]. All statistical analyses were performed using R software and Bioconductor.

### Functional enrichment analysis

Functional enrichment analysis at the Gene ontology (GO) and Kyoto encyclopedia of genes and genomes (KEGG) pathway levels were conducted to infer lncRNA function using the DAVID Bioinformatics Tool (https://david.ncifcrf.gov/, version 6.7) [84, 85], a widely used functional annotation tool that can extract the major biological significance among a gene set of interest. The results of enrichment analysis were obtained limited to GO terms in the “Biological Process” (GOTERM-BP-FAT) and KEGG pathway categories using the functional annotation clustering and functional annotation chart options with the human whole genome as background. The enriched GO terms and KEGG pathway with p-value <0.05 and enrichment score >1.0 were considered as potential function of prognostic lncRNAs. Significant GO terms with similar function were visualized as interaction networks using the Enrichment Map plugin in Cytoscape [86].

## SUPPLEMENTARY FIGURES AND TABLE




