# Supplementary material for: Comprehensive analysis of lncRNA expression profiles reveals a novel lncRNA signature to discriminate nonequivalent outcomes in patients with ovarian cancer
Source: Oncotarget. 2016 Apr 18;7(22):32433–48. doi: 10.18632/oncotarget.8653 (PMC5078024; doi:10.18632/oncotarget.8653)
Supplement: Supplementary file 1 [file oncotarget-07-32433-s001.pdf]

Comprehensive analysis of lncRNA expression profiles reveals a novel lncRNA signature to discriminate nonequivalent outcomes in patients with ovarian cancer

SUPPLEMENTARY FIGURES AND TABLE

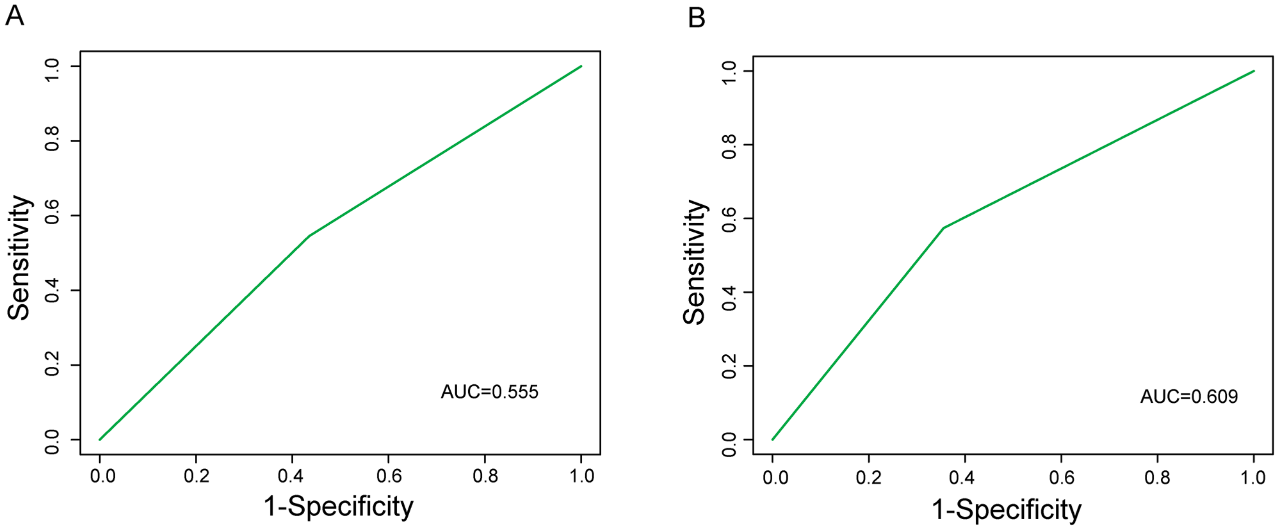

Supplementary Figure S1: The time-dependent ROC curves within 5 years for the eight-lncRNA signature in the validation cohort A. and in the entire TCGA cohort **B.**

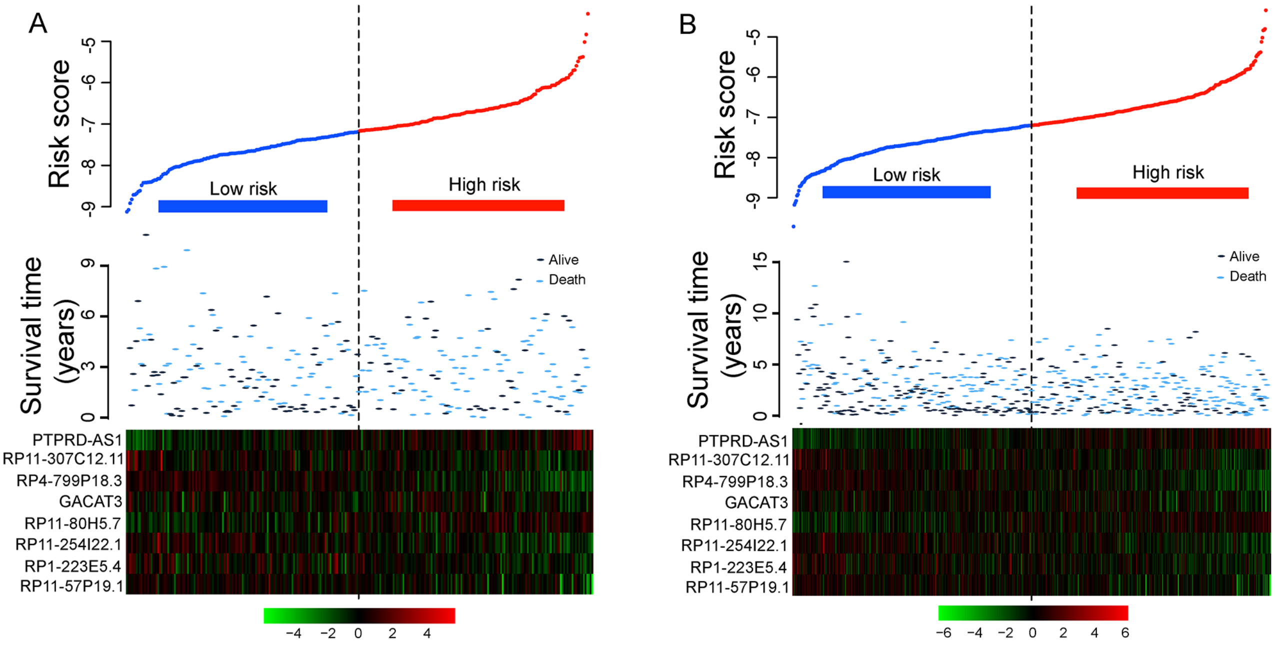

Supplementary Figure S2: The distribution of risk scores, survival status and lncRNA expression for patients in the validation cohort A. and in the entire TCGA cohort **B.**

Supplementary Table S1: Detailed clinical information of OvCa patients enrolled in this study.

See Supplementary File 1
